# Investigation of SARS-CoV-2 faecal shedding in the community: a prospective household cohort study (COVID-LIV) in the UK

**DOI:** 10.1186/s12879-021-06443-7

**Published:** 2021-08-09

**Authors:** Natasha Marcella Vaselli, Wega Setiabudi, Krishanthi Subramaniam, Emily R. Adams, Lance Turtle, Miren Iturriza-Gómara, Tom Solomon, Nigel A. Cunliffe, Neil French, Daniel Hungerford, on behalf of the COVID-LIV Study Group, Lance Turtle, Daniel Hungerford, Krishanthi Subramaniam, Roberto Vivancos, Mark Gabbay, Iain Buchan, Enitan D. Carrol, Miren Iturriza-Gómara, Tom Solomon, Nigel A. Cunliffe, Emily R. Adams, Carrol Gamble, Lynnette Crossley, Neil Joseph, Wega Setiabudi, Natasha Marcella Vaselli, Moon Wilton, Lee D. Troughton, Samantha Kilada, Katharine Abba, Victoria Simpson, John S. P. Tulloch, Lynsey Goodwin, Rachael Daws, Shiva Seyed Forootan, Susan Dobson, Rachel Press, Vida Spaine, Lesley Hands, Kate Bradfield, Carol McNally, Tracy Moitt, Silviya Balabanova, Chloe Donohue, Lynsey Finnetty, Laura Marsh, William Greenhalf, Dean J. Naisbitt, Victoria E. Shaw, Stephen Aston, Gareth Platt, Christopher Dunn, Paul J. Thomson, Monday Ogese, Sean Hammond, Kareena Adair, Liam Farrell, Joshua Gardner, Kanoot Jaruthamsophon, Serat-E Ali, Adam Lister, Laura Booth, Milton Ashworth, Katie Bullock, Benjamin W. A. Catterall, Terry Foster, Lara Lavelle-Langham, Joanna Middleton, William Reynolds, Emily Cass, Alejandra Doce Carracedo, Lianne Davies, Lisa Flaherty, Melanie Oates, Nicole Maziere, Jennifer Lloyd, Christopher Jones, Hannah Massey, Anthony Holmes, Nicola Carlucci, Vanessa Brammah, Yasmyn Ramos, Daniel Allen, Jane Armstrong, Debbie Howarth, Eve Wilcock, Jenna Lowe, Jayne Jones, Paula Wright, Iain Slack, Simone McLaughlin, Jessica Mason, Thomas Edwards, Claudia McKeown, Elysse Hendrick, Chris Williams, Rachel Byrne, Kate Buist, Gala Garrod, Sophie Owen, Ashley P. Jones, Efstathia Gkioni

**Affiliations:** 1 Department of Clinical Infection Microbiology and Immunology, Institute of Infection, Veterinary & Ecological Sciences, University of Liverpool, Liverpool, UK; 2 NIHR Health Protection Research Unit in Emerging and Zoonotic Infections at the University of Liverpool, Liverpool, UK; 3 Department of Tropical Disease Biology, Liverpool School of Tropical Medicine, Liverpool, UK; 4 Current address: Centre for Vaccine Innovation and Access, PATH, Geneva, Switzerland; 5 Walton Centre NHS Foundation Trust, Liverpool, UK; 6 NIHR Health Protection Research Unit in Gastrointestinal Infections at the University of Liverpool, Liverpool, UK

**Keywords:** SARS-CoV-2, COVID-19, Cohort study, Faecal shedding, Transmission, Community, Asymptomatic, Gastrointestinal

## Abstract

**Background:**

SARS-CoV-2 is frequently shed in the stool of patients hospitalised with COVID-19. The extent of faecal shedding of SARS-CoV-2 among individuals in the community, and its potential to contribute to spread of disease, is unknown.

**Methods:**

In this prospective, observational cohort study among households in Liverpool, UK, participants underwent weekly nasal/throat swabbing to detect SARS-CoV-2 virus, over a 12-week period from enrolment starting July 2020. Participants that tested positive for SARS-CoV-2 were asked to provide a stool sample three and 14 days later. In addition, in October and November 2020, during a period of high community transmission, stool sampling was undertaken to determine the prevalence of SARS-CoV-2 faecal shedding among all study participants. SARS-CoV-2 RNA was detected using Real-Time PCR.

**Results:**

A total of 434 participants from 176 households were enrolled. Eighteen participants (4.2%: 95% confidence interval [CI] 2.5–6.5%) tested positive for SARS-CoV-2 virus on nasal/throat swabs and of these, 3/17 (18%: 95% CI 4–43%) had SARS-CoV-2 detected in stool. Two of three participants demonstrated ongoing faecal shedding of SARS-CoV-2, without gastrointestinal symptoms, after testing negative for SARS-CoV-2 in respiratory samples. Among 165/434 participants without SARS-CoV-2 infection and who took part in the prevalence study, none had SARS-CoV-2 in stool. There was no demonstrable household transmission of SARS-CoV-2 among households containing a participant with faecal shedding.

**Conclusions:**

Faecal shedding of SARS-CoV-2 occurred among community participants with confirmed SARS-CoV-2 infection. However, during a period of high community transmission, faecal shedding of SARS-CoV-2 was not detected among participants without SARS-CoV-2 infection. It is unlikely that the faecal-oral route plays a significant role in household and community transmission of SARS-CoV-2.

**Supplementary Information:**

The online version contains supplementary material available at 10.1186/s12879-021-06443-7.

## Background

On January 30th 2020 the World Health Organisation (WHO) declared a public health emergency of international concern with regards to the spread of the novel virus severe acute respiratory syndrome coronavirus 2 (SARS-CoV-2), which cause COVID-19 [1].COVID-19 is a multisystem disease with the most commonly reported symptoms comprising fatigue, non-productive cough, dyspnoea and myalgia [2, 3]. Patients may also present with gastrointestinal symptoms including abdominal pain, diarrhoea, anorexia, nausea and vomiting [2–4].

Epidemiological studies have shown that transmission occurs mostly by respiratory droplets, but also via fomites and possibly via aerosols [3, 5]. However, there is increasing evidence to suggest that the faecal-oral route could also play a role in the transmission of SARS-CoV-2. Zhang et al. first isolated live SARS-CoV-2 virus from faecal samples; following inoculation of the stool suspension into Vero cells, the virus was observed under electron microscopy [6]. The presence of live virus in faecal specimens of SARS-CoV-2 infected patients has subsequently been confirmed in several studies, including from patients without gastrointestinal symptoms [7–9].

Studies in hospitalised patients have reported SARS-CoV-2 faecal shedding in up to 50% of cases [10]. A recent systematic review investigated the presence of viral RNA either in the faeces or in the intestinal cells of patients with a confirmed diagnosis of COVID-19 [11]. The systematic review included 27 studies, evaluating a total of 671 patients with laboratory-confirmed COVID-19, of whom 312 (46.5%) had a positive stool sample for viral RNA [11].

There are no published studies investigating faecal shedding in an asymptomatic or pauci-symptomatic community-based population. We have conducted a community household cohort study in Liverpool City Region, UK since July 2020, known as the COVID-LIV Study [12]. The incidence of SARS-CoV2 infection in Liverpool reached a weekly rate of 659 per 100,000 population in early-mid October 2020, with a positivity rate of 18.1% [13]. This provided an opportunity to investigate faecal shedding of SARS-CoV2 in a high incidence community setting, prior to vaccine introduction in order to better understand the role of faecal-oral transmission in disease spread, to subsequently inform the implementation of appropriate public health measures. We obtained serial stool samples from respiratory positive SARS-CoV-2 cases and undertook a two-point prevalence stool sampling investigation among COVID-LIV households.

## Methods

### Study design

The COVID-LIV Study is an observational cohort study recruiting households in the Liverpool City Region to investigate household transmission of SARS-CoV-2. Selection and recruitment of participants, along with detailed study methodology, has been described previously [12]. Briefly, participants were recruited from an established household health survey undertaken by the NIHR Collaboration for Leadership in Applied Health Research and Care (CLAHRC, now Applied Research Collaboration, ARC) and through GP surgeries and local media advertisements [14]. Enrolment of households began in July 2020; participants underwent baseline and three-monthly follow-up serological and immunological investigations, together with a weekly combined self-administered nasal/throat swab for SARS-CoV-2, for a total of 12 weeks from enrolment. All participants were invited to complete a weekly electronic questionnaire detailing their social interactions and the presence of any symptoms of illness (Additional file 1).

### Stool sampling

Participants that tested positive for SARS-CoV-2 on combined nasal/throat swabs were asked to provide a stool sample within approximately 3 days of the positive test result and 14 days later. In mid-October 2020 all households were invited by email to undertake optional stool sampling at two time points, once in October 2020 and once in November 2020. Those participants who indicated via email that they would be willing to provide a stool sample were provided with stool sampling kits. A courier picked up the stool samples within 24 h of the stool sample being taken and returned them to the laboratory at the University of Liverpool. Upon receipt, the stool samples were stored at − 80 °C.

### Laboratory testing

#### Nasal/throat swabs

Nasal/throat swabs were collected using eswab (Copan Diagnostics, USA) and stored at 4 °C overnight. SARS-CoV-2 RNA was extracted using the *Quick*-DNA/RNA Viral MagBead extraction kit (Zymo Research Corp. USA).

#### Stool samples

The samples were thawed and approximately 200–400 mg of faecal material was resuspended in 1.60 ml phosphate buffered saline (PBS) and stored at − 80 °C until RNA extraction was undertaken. Following thawing, the faecal suspensions were vortexed for 30 s and 300 μl was mixed 1:1 with 300 ul DNA/RNA Shield (Zymo Research Corp. USA). Samples were centrifuged at 5000 rpm for 3 min to pellet debris and 400ul of the supernatant was taken for RNA extraction, using the *Quick*-DNA/RNA Viral MagBead extraction kit (Zymo Research Corp. USA).

#### SARS-CoV-2 RNA detection

SARS-CoV-2 RNA was detected using the Genesig® Real-Time PCR COVID-19 (CE) assay (Primerdesign Ltd., UK), which targets the orf1 ab genome region, on a Rotor-Gene Q (Qiagen, Germany). The internal extraction control (IEC) template from the Genesig® Real-Time PCR kit (Primerdesign Ltd., UK) was added to each sample prior to extraction according to manufacturer’s instructions, and detected using RT-PCR. If the IEC failed the sample was re-extracted and again tested by RT-PCR. Each RT-PCR run also included a positive template control from the Genesig® Real-Time PCR kit (Primerdesign Ltd., UK). Samples with a cycle threshold (Ct) value of below 37 were considered positive. Samples with a Ct value between 37 and 40 were considered indeterminate and were retested in triplicate; samples were considered positive if two out of three replicates had a Ct value below 40.

### Participant variables

Participants provided demographic details, including date of birth, sex and postcode of residence. Postcode of residence was used to assign socio-economic status using English Index of Multiple Deprivation (IMD). For participants who tested positive for SARS-CoV-2 on nasal/throat swab, self-reported clinical data were obtained including symptoms at the time of positive test, medications and presence of co-morbidities.

### Data analysis

Characteristics of the study cohort who provided a stool sample were compared with those that did not, by using a χ2 test or Fisher’s exact test for categorical variables and Wilcoxon rank sum test for continuous variables. Exact binomial 95% confidence intervals (CIs) for proportions were calculated for SARS-CoV-2 positive participants. All analyses were conducted using R version 4.0.3 (R Core Team, Vienna, Austria).

## Results

### Study population

**Fig. 1:**
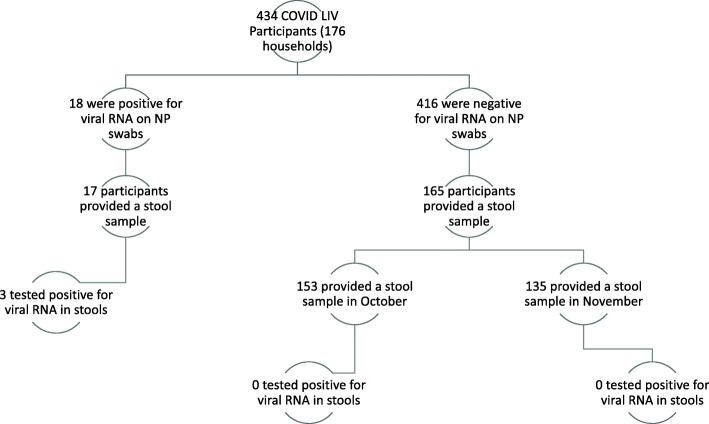
Stool samples and SARS-CoV-2 PCR test results among COVID-LIV participants. (123 participants took part in the stool sampling at both time points)

A total of 176 households including 434 participants were enrolled in the COVID-LIV Study between 9th July and 30th September 2020. Median age of participants was 46.0 (IQR: 24.3–63.0) and 53.7% (222/434) identified as female. Of the 176 enrolled households, 17.6% (31/176) were one person households, 46% (81/176) were two person households, 14.8% (26/176) were three person households and 21.6% (38/176) four of more person households. Among enrolled participants, 18/434 (4.2%: 95% CI 2.5–6.5) tested positive for SARS-CoV-2 on nasal/throat swab during the 12 week active follow-up period. Of these 18 SARS-CoV-2 positive cases, two were from the same household but tested positive > 6 weeks apart, 55.6% (10/18) were female and the median age was 42 years (range 10–72 years); 17 provided at least one stool sample (Fig. 1). Among 40% (165/416) of SARS-CoV-2 nasal/throat swab negative participants who provided a stool sample within the two-point prevalence sampling, 53.9% (89/165) were female and the median age was 58 years (range 1–86 years). Participants that took part in the prevalence stool sampling were more likely to be older (*p* < 0.001) and from less socioeconomically deprived IMD deciles (*p* < 0.001) than those that did not take part (Table 1) There was no difference in sex between the two groups of participants (*p* = 0.912; Table 1). A total of 153 participants provided a sample during the October 2020 sampling period and 135 in the November 2020 sampling period; 123 participants provided stools samples at both time points (Fig. 1).

**Table 1:** Characteristics of COVID-LIV participants

Characteristics	SARS-CoV-2 nasal/ throat swab negative participants				SARS-CoV-2 nasal/ throat swab positive participants
	No stool provided	Stool provided	Overall	***P*** value	
	(*N* = 251)	(*N* = 165)	(*N* = 416)		(*N* = 18)
**IMD**				< 0.001	
1 (most deprived)	70 (27.9%)	25 (15.2%)	95 (22.8%)		5 (27.8%)
2	32 (12.7%)	15 (9.1%)	47 (11.3%)		3 (16.7%)
3	15 (6.0%)	14 (8.5%)	29 (7.0%)		< 3
4	30 (12.0%)	16 (9.7%)	46 (11.1%)		< 3
5	22 (8.8%)	15 (9.1%)	37 (8.9%)		< 3
6	24 (9.6%)	25 (15.2%)	49 (11.8%)		< 3
7	12 (4.8%)	15 (9.1%)	27 (6.5%)		< 3
8	18 (7.2%)	23 (13.9%)	41 (9.9%)		< 3
9	17 (6.8%)	3 (1.8%)	20 (4.8%)		< 3
10 (least deprived)	11 (4.4%)	14 (8.5%)	25 (6.0%)		< 3
**Age**					
Median IQR [Min, Max]	15/39/57 [1, 82]	42/58/66 [1, 86]	24/46/63 [1, 86]	< 0.001	26/42/61 [10, 72]
**Sex**					
Female	134 (53.4%)	89 (53.9%)	223 (53.6%)	0.912	10 (55.6%)
Male	117 (46.6%)	76 (46.1%)	193 (46.4%)		8 (44.4%)

The *P* value compares those who provided a stool sample and those who did not in the SARS-CoV-2 nasal/throat swab negative population

IMD refers to the English Index of Multiple Deprivation deciles, assigned based on patient postcode

IQR refers to the interquartile range

< 3 refers to numbers suppressed to prevent participant disclosure

### SARS-CoV-2 nasal/throat swab PCR positive participants

**Fig. 2:**
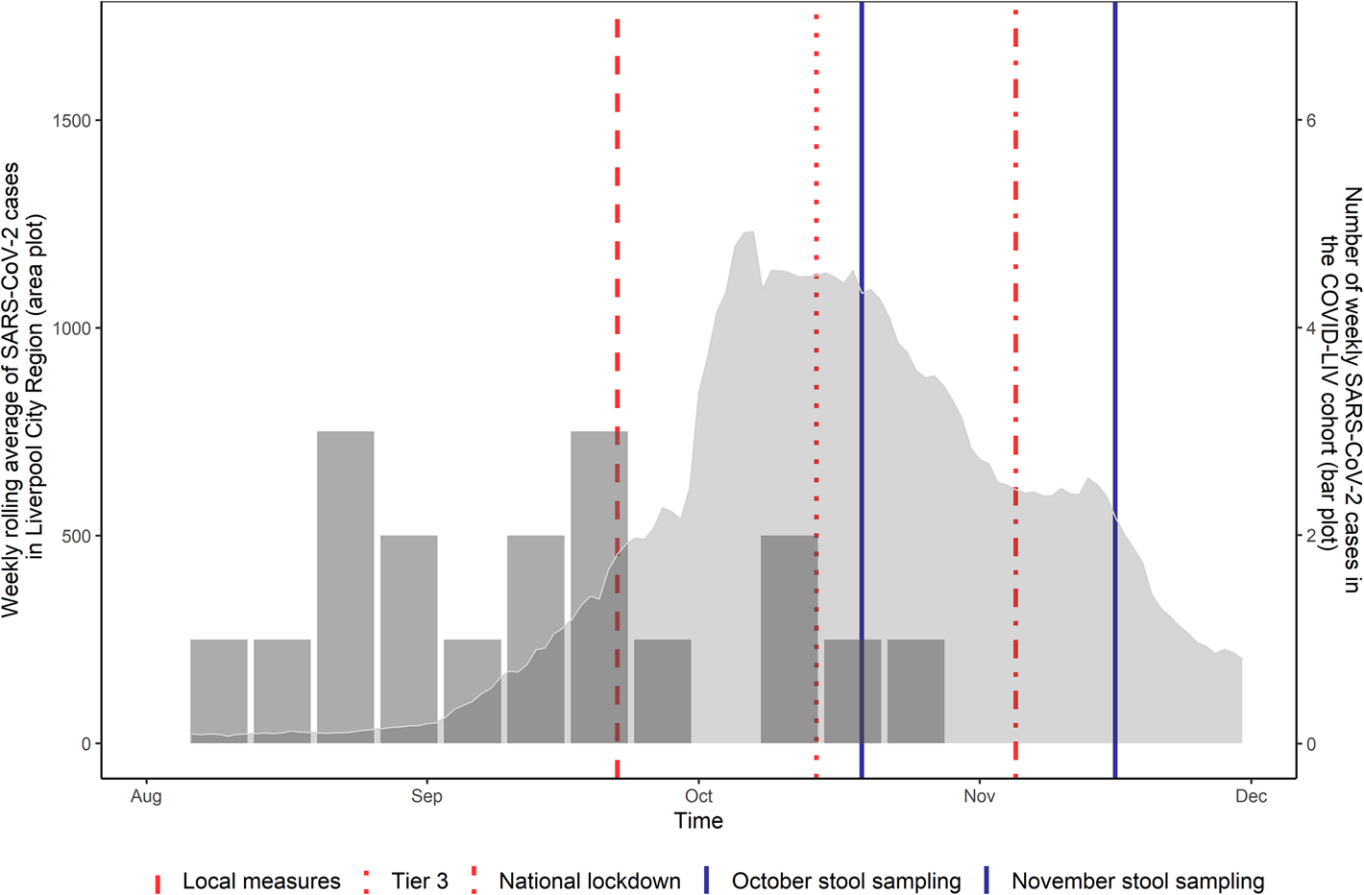
Background rate of SARS-CoV-2 cases in the Liverpool City Region in relation to the timing of COVID-LIV cases, stool sampling and public health measures. (SARS-CoV-2 case data for the Liverpool City Region adapted from https://coronavirus.data.gov.uk/details/download [accessed 09/12/2020])

All PCR positive cases occurred between August and October 2020, with the majority (77.8%; 14/18) of the cases occurring before the peak of SARS-CoV-2 infection in the Liverpool City Region (Fig. 2). Five of the cases (27.8%) tested positive for SARS-CoV-2 on two or more consecutive nasal/throat swabs. Cases were from a variety of household sizes ranging from one to four occupants. Six cases (33.3%) were asymptomatic and three cases (16.7%) reported gastrointestinal symptoms including diarrhoea, nausea and vomiting. None of the cases was hospitalised in the 2 weeks following the first nasal/throat swab positive result. All cases reported no co-morbidities.

#### SARS-CoV-2 faecal shedding among nasal/throat swab PCR positive participants

Three of the 17 participants who provided a stool sample had SARS-CoV-2 RNA detected (18%: 95% CI 4–43%). The three participants with faecal shedding included a male aged 15–19 years (four person household; two household members aged 50–54 years and one aged 15–19 years), a female aged 15–19 years (two person household; other household member aged 55–59 years) and 50–54 year old male (three person household; other household members aged 50–54 years and 20–24 years); none reported gastrointestinal symptoms. None of the three faecal shedders took any regular medications. The Ct values for the stool samples ranged from 20.00–33.46; of the three participants who faecally shed viral RNA, two had among the lowest Ct values (highest viral load) on the nasal/throat swabs (16.40 and 19.18), and one participant tested positive on repeat nasal/throat swabbing (Table 2). Two out of the three participants had detectable virus in stool beyond the time that respiratory samples tested negative for viral RNA (Table 2). One participant tested positive for viral RNA in the stool at 16 days, and the other participant at 21 days after a nasal/throat swab positive test for SARS-CoV-2 virus; they continued to shed in their stools for three and 7 days respectively after testing PCR negative on nasal/ throat swab. There was no transmission within the households of those who tested positive for viral RNA in stool.

**Table 2:** Features of SARS-CoV-2 nasal/throat swab PCR positive COVID-LIV participants

ID^a^	Nasal/throat swab positive (first test is day 1)	Nasal/throat Ct values	Nasal/throat Swab negative (days since first positive nasal/throat swab)	Stool samples (days since first positive nasal/throat swab)	Stool positive (days since first positive nasal/throat swab)	Stool Ct values
**a-002**	1	35.00	7	5, 49, 78	Negative	N/A
**b-001**	1	35.49	15	8, 23, 71, 86	Negative	N/A
**c-002**	1	35.69	14	6, 21, 69, 98	Negative	N/A
**d-002**	1, 6	33.87, 35.11	15	8, 16	8, 16	20.00, 33.03
**e-002**	1	35.28	6	7,20	Negative	N/A
**f-001**	1	35.01	7	8, 18	Negative	N/A
**g-001**	1	34.41	7	6, 20, 28	Negative	N/A
**h-003**	1, 5, 7	22.13, 32.65, 28.05	14	6	Negative	N/A
**i-002**	1	36.79	6	7, 15	Negative	N/A
**j-001**	1	35.60	7	4, 15	Negative	N/A
**k-002**	1	27.07	6	6, 18	Negative	N/A
**l-002**	1	36	12	6, 13, 23	Negative	N/A
**m-003**	1, 8, 14	32.90, 28.52, 34.07	21	6, 19	Negative	N/A
**n-003**	1	30.78	6	6, 17, 34, 61	Negative	N/A
**o-003**	1, 7	16.40, 35.48	14	21	21	23.66
**p-002**	1, 7	19.18,33.14	NA	4	4	33.46
**q-001**	1	30.62	6	7, 14	Negative	N/A
**r-003**	1	31.70	6	NA	N/A	N/A

^a^Random anonymous ID

### Faecal shedding of SARS-CoV-2 among nasal/throat swab PCR negative participants

None of the 165 participants that tested negative for SARS-CoV-2 RNA in the upper respiratory tract had viral RNA detected in their stool samples in either October or November 2020.

## Discussion

To our knowledge this is the first reported community-based study investigating faecal shedding of SARS-CoV-2. The overall rate of faecal shedding in this community was low; while 3/17 participants who tested positive for SARS-CoV-2 on nasal/throat swab showed evidence of faecal shedding, 0/165 participants that were negative for SARS-CoV-2 on nasal/throat showed evidence of viral RNA in their stools when sampled over two time points.

In comparison to the relatively low rate of faecal shedding among respiratory PCR positive subjects reported here, studies conducted in hospital settings have reported faecal shedding in up to 50% of patients with COVID-19 [10]. This could potentially be due to differences in the severity of symptoms, viral load in the respiratory tract, or in the propensity of the virus to disseminate, which post-mortem evidence suggests happens in patients who have died from severe COVID-19 [15]. However, our study showed that participants with both high and low Ct values in respiratory material shed the virus faecally. Moreover, a recent study showed no difference in the duration of viral shedding, or in SARS-CoV-2 load, in stool samples among patients with mild or severe disease [16]. This highlights that other factors other than viral load are likely to be involved in the faecal shedding of SARS-CoV-2.

Faecal shedding in this study was not associated with the presence of gastrointestinal symptoms. Previous studies of faecal shedding in patients who were asymptomatic or had mild symptoms of SARS-CoV-2 infection also demonstrated a lack of association between faecal shedding and gastrointestinal symptoms [4, 17–20]. However, several hospital-based studies found that gastrointestinal symptoms, in particular diarrhoea, were associated with faecal shedding [21–23]. Moreover, those with gastrointestinal symptoms had a longer duration between symptom onset and viral clearance [21]. A recent study investigated the intestinal microbiota in SARS-CoV-2 hospitalised patients with GI manifestations. This study found that patients with prolonged GI manifestations had a reduction in the diversity and richness in their microbiota and prolonged viral clearance [24]. Studies report that the relative abundance of the microbiota *Coprobacillus, Clostridium ramosum*, and *Clostridium hathewayi* positively correlated to COVID-19 severity [25]. Furthermore, studies have shown that ACE-2 receptor is the entry point for SARS-CoV-2 virus into the gastrointestinal tract [26]. The interaction between the ACE-2 receptor and SARS-CoV-2 can lead to receptor dysregulation, intestinal inflammation and the manifestation of gastrointestinal symptoms [25]. Differences in the expression of the ACE-2 receptor in the gastrointestinal tract could explain differences in faecal shedding and gastrointestinal symptoms among individuals with SARS-CoV-2 infection.

Two out of three participants that had viral RNA detected in their stools demonstrated persistent faecal shedding despite negative nasal/throat swabs for SARS-CoV-2. Previous studies in hospitalised patients have demonstrated prolonged faecal shedding for up to 5 weeks after respiratory samples were negative for viral RNA, including one study which showed that 80% of children had persistent positive real time RT- PCR test of rectal swabs [19, 27, 28].

Persistent faecal shedding of SARS-CoV-2 may potentially allow faecal-oral transmission. However, within our study there was no transmission of infection within the households of participants who had detectable viral RNA in their stools. Furthermore, in the two-point prevalence stool sampling none of the participants had detectable SARS-CoV-2 RNA in stool, despite sampling being undertaken during a period of high transmission of SARS-CoV-2 in the community. Together, our data do not suggest a significant role for faecal-oral transmission in the community. Additionally, our findings have implications for the utility of rectal swabs for COVID-19 diagnosis and public health surveillance, and for the use of wastewater for surveillance of SARS-CoV-2 [8, 29]. A number of the participants, particularly those younger and from more socioeconomically deprived neighbourhoods, did not provide faecal samples. This further suggests that the use of rectal swabs could produce partial, and systematically biased data.

### Limitations

Participant engagement in the two-point prevalence stool sampling was low. Only 40% (165/416) of enrolled, respiratory PCR negative participants provided a stool sample; participants providing a stool sample were slightly older and less deprived than the remainder of the COVID-LIV population. This highlights the difficulty of undertaking stool surveillance in the community, even among our research-engaged study population. Additionally, as faecal shedding appears more common in children compared with adults, we may have underestimated shedding in the respiratory negative participants [27, 28]. We also did not obtain enough serial stool samples to determine cessation of faecal shedding. Furthermore, for mucosal pathogens such as SARS-CoV-2, relating PCR detection of viral nucleic acid in clinical samples to infectiousness remains problematic. Finally, during the study period there were few strains of the SARS-CoV-2 virus in circulation. Since December 2020, there has been an increase in the alpha (B.1.1.7; first detected in the UK) variant in the UK which has spread globally and from May 2021 the delta (B.1.617.2; first detected in India) variant [30, 31]. As new strains emerge it is important to investigate the propensity of these new variants to be associated with faecal shedding, and the consequent role of faecal-oral transmission in virus spread.

## Conclusion

In this community household cohort study, faecal shedding of SARS-CoV-2 viral RNA was detected among respiratory positive participants. However, in two-point prevalence stool sampling conducted during an intense period of community transmission, we did not detect SARS-CoV-2 viral RNA in the stool of SARS-CoV-2 respiratory negative participants. Our study demonstrates that faecal shedding of SARS-CoV-2 is unlikely to play a significant role in transmission of infection in households and in the community.

## Supplementary Information

**Additional file 1.**

## Data Availability

After completion of the full COVID-LIV study de-identified participant data will be made available to others for meta-analysis upon request following Study Steering Group discussion and signing of a data access agreement. Requests for access to data should be made to the corresponding author via the corresponding email given.

## Abbreviations

ARC: Applied Research Collaboration
CI: Confidence interval
IMD: Index of Multiple Deprivation
NHS: National Health Service
PCR: Polymerase Chain Reaction
RNA: Ribose Nucleic Acid
RT-PCR: Reverse transcription Polymerase Chain Reaction
SARS-CoV-2: Severe acute respiratory syndrome coronavirus 2
SD: Standard deviation
UK: United Kingdom
WHO: World Health Organisation
