# Supplementary material for: Investigation of SARS-CoV-2 faecal shedding in the community: a prospective household cohort study (COVID-LIV) in the UK
Source: BMC Infect Dis. 2021 Aug 9;21:784. doi: 10.1186/s12879-021-06443-7 (PMC8352155; doi:10.1186/s12879-021-06443-7)
Supplement: Supplementary file 1 — Additional file 1. [file 12879_2021_6443_MOESM1_ESM.pdf]

COVID-LIV Form 4 Weekly Follow-Up

COVID-LIV Study

Household ID Number ☐☐☐

Date of timepoint: d d m m 2 0 y y

Timepoint of follow up Week 1 ☐ Week 2 ☐ Week 3 ☐ Week 4 ☐ Week 5 ☐

Week 6 ☐ Week 7 ☐ Week 8 ☐ Week 9 ☐ Week 10 ☐ Week 11 ☐ Week 12 ☐

Who is completing this form?

Are you completing this form about yourself? Yes ☐ → If yes, please provide your own Household Member ID Number: ☐☐☐

No ☐ → If no, please provide the Household Member ID Number of the person you are completing on behalf of: ☐☐☐

→ In no, please indicate which type of informant you are:

- Parent ☐
- Spouse, sibling or child ☐
- Grandparent, Grandchild or other relative ☐
- Non-relative ☐

Questions about the household individual start here.

Is the household member still present? Yes ☐ No ☐ If no, please select the reason below

L Left, **H** Hospitalised, **D** Deceased, **O** other, **NK** Not Known ☐ If other, please specify ______________________

Section 1 - Work undertaken outside of the home

During the last week, did you undertake paid employment?	Yes	☐	No	☐
During the last week, did you undertake non paid work?	Yes	☐	No	☐
During the last week, did you leave the house to work?	Yes	☐	No	☐

If yes how many times? ☐☐ Estimate how many hours ☐☐

COVID-LIV Form 4 Weekly Follow-Up

Household ID Number ☐☐☐

Section 2 - Medical History

Are you currently ill? Yes ☐ No ☐ If no, skip to Section 3

Cough	Yes ☐	No ☐	Duration of days:	☐☐☐
Fever	Yes ☐	No ☐	Duration of days:	☐☐☐
Sore throat	Yes ☐	No ☐	Duration of days:	☐☐☐
Headache	Yes ☐	No ☐	Duration of days:	☐☐☐
Breathing difficulty	Yes ☐	No ☐	Duration of days:	☐☐☐
Diarrhoea	Yes ☐	No ☐	Duration of days:	☐☐☐
Rash	Yes ☐	No ☐	Duration of days:	☐☐☐
Chest pain	Yes ☐	No ☐	Duration of days:	☐☐☐
Disturbance or loss of smell (Anosmia)	Yes ☐	No ☐	Duration of days:	☐☐☐
Abdominal pain	Yes ☐	No ☐	Duration of days:	☐☐☐
Muscle aches & pains	Yes ☐	No ☐	Duration of days:	☐☐☐
Other - Specify ______________________	Yes ☐	No ☐	Duration of days:	☐☐☐
Other - Specify ______________________	Yes ☐	No ☐	Duration of days:	☐☐☐
Other - Specify ______________________	Yes ☐	No ☐	Duration of days:	☐☐☐

Section 3 - Recent movement

In the past week, excluding work undertaken outside of the home, have you been out of the house?

Yes ☐ No ☐ If no, skip to Section 4

If yes please complete the number of times for each activity below, please provide a ‘0’ if applicable.

If you left the house 1 or more times to complete an activity please provide the number of hours you spent outside the house

**Reason**	Number of times (estimate)	Number of hours (estimate)
Shopping	☐☐	☐☐
Exercise	☐☐	☐☐
School/Taking children to school	☐☐	☐☐
Other, specify reason below		
1) ______________________	☐☐	☐☐
2) ______________________	☐☐	☐☐
3) ______________________	☐☐	☐☐

COVID-LIV Form 4 Weekly Follow-Up

Household ID Number ☐☐☐

Section 4 - Contact in house

In the past 7 days have you eaten meals with other members of the household? Yes ☐ No ☐

If yes, on how many out of the 7 days? ☐

If yes, what is the maximum number of people you have had a meal with? ☐☐

In the past 7 days have you watched television/gaming with other members of the household? Yes ☐ No ☐

If yes, on how many out of the 7 days? ☐

If yes, what is the maximum number of people you have watched with? ☐☐

In the past 7 days have you slept in the same room as anyone else in the household? Yes ☐ No ☐

If yes, do you sleep in the same bed? Yes ☐ No ☐

Section 5 - COVID-19 in the household?

Other than yourself is there a case of COVID-19 in the household? Yes ☐ No ☐ If yes, please answer the following.

Since the ill household member began to have symptoms, on average per day how many hours have you spent in the same room ? ☐☐

Since the day symptoms began for the ill household member have you:

Slept in the same room as the household member who is ill ?	Yes ☐	No ☐
Hugged or embraced the household member who is ill ?	Yes ☐	No ☐
Assisted the household member who is ill with the toilet (including changing their nappy) ?	Yes ☐	No ☐
Assisted the household member who is ill with washing ?	Yes ☐	No ☐
Assisted the household member who is ill with walking ?	Yes ☐	No ☐
Failed to wash hands after caring for household member who is ill ?	Yes ☐	No ☐
Shared a hand towel with household member who is ill ?	Yes ☐	No ☐
Worn a face mask in the house ?	Yes ☐	No ☐
Eaten food prepared by the household member who is ill ?	Yes ☐	No ☐

Section 6 - Samples

Nasal swab taken Yes ☐ No ☐
